# Dual microelectromembrane extraction as a tunable platform for the determination of antioxidant compounds with varied hydrophobicity in oral bioaccessibility assays of food commodities: a proof of concept

**DOI:** 10.1007/s00216-025-05744-z

**Published:** 2025-02-01

**Authors:** Ali Sahragard, Carlos Pagan-Galbarro, David J. Cocovi-Solberg, Manuel Miró

**Affiliations:** 1 FI-TRACE Group, Department of Chemistry, Faculty of Science, University of the Balearic Islands, Illes Balears, Carretera de Valldemossa Km 7.5, 07122 Palma, Spain; 2 Department of Chemistry, Institute of Analytical Chemistry, University of Natural Resources and Life Sciences, Muthgasse 18, 1190 Vienna, Austria

**Keywords:** Dual microelectromembrane extraction, Flow analysis, Green analytical chemistry, Polyphenolic acids, Oral bioaccessibility tests

## Abstract

An automatic millifluidic dual microelectromembrane extraction (D-µEME) method as a front-end to HPLC-UV-Vis is herein proposed for the first time to facilitate the matrix clean-up of relatively polar polyphenolic acidic (PPA) antioxidants with a relatively broad range of lipophilicity (log*P* from −0.27 to 2.14) from simulated gastric extracts of oral bioaccessibility tests. The flow setup is amenable to handle microliter volumes of two distinct organic phases along with donor and acceptor phases unsupervised, conduct in-tube D-µEME in parallel without supporting membranes, and mix the two acceptor phases automatically prior to online HPLC-UV-Vis. The target antioxidants involve gallic acid, chlorogenic acid, 4-hydroxybenzoic acid, caffeic acid, and *trans*-cinnamic acid. Various solvents are explored to investigate their compatibility for simultaneous D-µEME, including 1-pentanol, 1-hexanol, 1-heptanol, 1-octanol, and 1-nonanol, as well as deep eutectic solvents, e.g., thymol/6-methyl coumarin, and ionic liquids as additives to alcohols. Notably, 1-pentanol and 1-octanol exhibit the best performances in extracting the most polar (gallic acid and chlorogenic acid) and the least polar analytes (*trans*-cinnamic acid), respectively, notwithstanding both solvents are amenable to retrieve analytes with medium hydrophobicity (4-hydroxybenzoic acid and caffeic acid). The effects of the voltage, the extraction time, and the sample ionic strength on the extraction recoveries are also investigated in detail. Under the selected D-µEME conditions, the overall linear ranges span from 1.25 to 80 mg/L, with limits of detection ranging from 0.2 to 3.3 mg/L. The flow-based D-µEME is resorted to oral bioaccessibility assays in the gastric phase of the target compounds from eggplant, blueberry, and coffee bean extracts, with relative extraction recoveries ranging from 71.5 to 133.5%.

**Graphical abstract:**

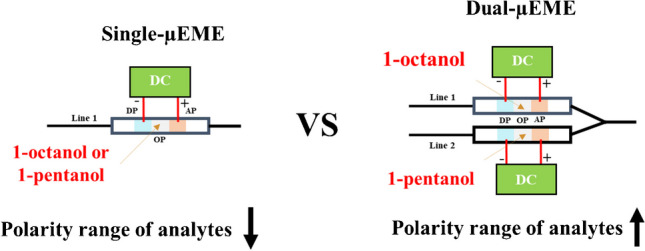

**Supplementary Information:**

The online version contains supplementary material available at 10.1007/s00216-025-05744-z.

## Introduction

Polyphenolic acids (PPAs) are categorized as derivatives of benzoic acid (BA) or derivatives of cinnamic acid based on their structure [1]. In the case of the former, PPAs consist of a benzene ring bonded to a carboxylic group, whereas the benzene ring is bonded to a propenoic acid moiety in the latter. The antioxidant, anti-tumor, anti-inflammatory, and immunomodulatory properties of dietary polyphenols have shown to play a role in safeguarding humans against various degenerative diseases, such as cancer and cardiovascular diseases, while helping improve gut health, blood pressure, lipid profiles, and insulin resistance [2–4]. In order to get insight into the actual role of polyphenols on the human health, the amount of polyphenols consumed in the diet and their bio-accessibility/availability should be studied. Most epistemological research has been concentrated on the digestion and intestinal absorption. This is reinforced by the fact that prevalent polyphenols in food might not be the most bioactive, nor the most bioaccessible/available [3, 5].

Total phenolic and antioxidant activity have been traditionally measured by spectrophotometric techniques involving the Folin-Ciocalteu method; however, the lack of method’s selectivity could give rise to false positive results [3, 6]. For this reason, there is a quest for chromatographic methods that are able to properly evaluate the individual phenolic content of different food commodities or beverages and their in vitro oral bioaccessibility [3]. Due to the potential matrix interference from the gastrointestinal extracts containing digestive enzymes, high salinity, and elevated acidity, direct injection of gastrointestinal fluids to the analytical instruments for detection of the bioaccessible PPAs can be detrimental, and generally many fold dilutions are necessary, compromising the method’s sensitivity. Therefore, there is a quest of implementing reliable sample preparation and clean-up steps in line with the principles of green chemistry to mitigate these effects. Unfortunately, to the best of our knowledge, there is no report on modern, automated, and miniaturized sample preparation methods for determination of bioaccessible PPAs in gastrointestinal fluids, although several microextraction approaches, such as single-drop microextraction [7], solid-phase microextraction [8], hollow fiber liquid-phase microextraction [9], ultrasonic-assisted liquid-liquid microextraction (LLME) [10], in-vial LLME [11], and electromembrane extraction (EME) [12, 13], as applied to food commodities have been reported in the literature.

Among the above microextraction methods, EME has emerged as an appealing sample treatment approach in the last decades. In EME, analytes migrate from an aqueous donor phase (DP), which could be a standard or real sample, to an acceptor phase (AP) through a supported (SLM) or non-supported liquid membrane [14–17]. In the case of extraction of antioxidant compounds [12, 13], sustainable methods capitalized upon gel-based EME and chitosan-based membranes should be underscored. However, all of these methods entail manual extraction and detection procedures, and are unable to miniaturize and automate the analytical procedure. Additionally, they did not cope with the impact of elevated ionic strength in the sample matrices, which is most relevant for their extension to the analysis of body fluids. High ionic strength typically disrupts the three phases in EME, leading to the failure of the extraction process because of the high electrical currents across the membrane [18]. Non-supported liquid membrane-based EME, called micro-EME (µEME), is an alternative to the more conventional SLM-based EME [19, 20]. µEME is performed by sandwiching a plug of organic solvent/phase (OP) between plugs of DP and AP [21]. The volume/thickness and the in-tube arrangement of these plugs can be precisely defined and performed using advanced flow systems, such as sequential injection analysis (SIA) setups [22]. It is worth mentioning that the development of SIA has given rise to the launching of pressure-driven meso/millifluidic platforms that are entirely computer-controlled, facilitating automatic sample preparation [23–25]. Additionally, the naked eye monitoring of the µEME phases is easier compared to SLM-EME due to the (semi)transparency of tubing used in the SIA-EME units [21]. Previously, µEME has been exploited in two [26, 27], three [28, 29], or five [30]-phase formats to address various analytical challenges. However, to the best of our knowledge, no automatic flow system has been reported to date for simultaneous dual μEME (D-µEME) of a class of compounds with the same charge but distinct polarity. The use of μEME for clean-up of gastrointestinal fluids has also not been described in the literature as of yet.

To tackle the above gaps identified in the literature, an automated SIA platform equipped with a fluidic D-µEME unit is herein fully leveraged as a front-end to on-line HPLC-UV-Vis for the unsupervised determination of the gastric bioaccessibility of PPAs with varied polarity, including gallic acid (GA), chlorogenic acid (ChA), 4-hydroxybenzoic acid (4-OH-BA), caffeic acid (CA), and *trans*-cinnamic acid (CiA). Investigation of the effect of the organic solvents, voltage, time, and ionic strength on the extraction efficiency of the model target analytes was comprehensively conducted with further applicability to oral bioaccessibility tests of food commodities, including eggplant, blueberry, and coffee bean extract.

## Experimental section

### Chemicals and oral bioaccessibility tests

All reagents and chemicals were of the analytical grade, GA (LogP 0.72), ChA (LogP − 0.27), 4-OH-BA (LogP 1.33), CA (LogP 1.53), CiA (LogP 2.14), and Aliquat®336 were obtained from Merck KGaA (Darmstadt, Germany). Pepsin, acetic acid, hydrochloric acid, sodium chloride, and sodium hydroxide were also purchased from Merck KGaA. 1-Octanol, methanol, 1-hexanol, 1-heptanol, 1-pentanol, thymol, and 6-methyl coumarin were obtained from Fisher Scientific (Madrid, Spain). A deep eutectic solvent (DES) was prepared by mixing thymol and 6-methyl coumarin in a 2:1 molar ratio [31]. Daily working standard solutions were prepared by diluting stock solutions (6000 mg/L) of target analytes in methanol with highly pure deionized water obtained from a Milli-Q system (18.2 MΩ·cm, Millipore, Molsheim, France). It is important to mention that certain PPAs, such as CiA, possess very low solubility in water. Hence, a constant concentration of 7.5% (v/v) methanol was maintained in all working standards and real samples to ensure data reliability across all experiments.

To prevent salt-dependent µEME results, the conductivities of the standards were adjusted with 2 mol/L NaCl to match that of the gastric fluid (adjusted at pH 7.0 before analysis) at room temperature (25 °C). Conductometric measurements were performed using a COND 7 + conductometer equipped with the COND Cell model 2301 T (XS Instruments, Carpi, Italy). A calibration curve ranging from 0 to 200 mmol/L NaCl solutions was obtained with the equation and the determination coefficient (*R*^2^) of conductivity (µS/cm) = 71.7 [NaCl, mmol/L] + 151.2 and 0.9986, respectively. The conductivity of the pH 7.0 adjusted gastric fluid with NaOH prior to µEME was determined to be 8360 µS/cm at 25 °C, which was equivalent to that of 114.5 mmol/L NaCl. Therefore, 120 mmol/L NaCl was added to all standards as a matrix-matched modifier in preliminary investigations of experimental SIA-µEME parameters.

The physiologically based extraction medium to simulate the human gastric fluid was adapted from the United States Pharmacopeia (USP) specifications [32] by dissolving a metered amount of pepsin (3.2 g) with an activity of 0.7 USP unit/mg in 0.7 mL concentrated HCl before making up to 1 L (final pH of ca. 1.4). This solution was transferred to a 2-L beaker and paddle-stirred with a speed of 500 rpm for 2 h at 37 °C. The real samples consisted of either 1 capsule of green coffee extract or 100 g of blueberry or eggplant purchased from the local market (Palma, Spain) and were subjected to the physiologically relevant extraction test using 1000 mL of the USP gastric fluid. Following gastric digestion, the pH of the samples was adjusted to 7.0 using a saturated NaOH solution. Samples were then filtered through polyvinylidene fluoride syringe filters (0.45 µm), prior to the D-µEME-HPLC-UV-Vis analysis.

### Equipment

**Fig. 1:**
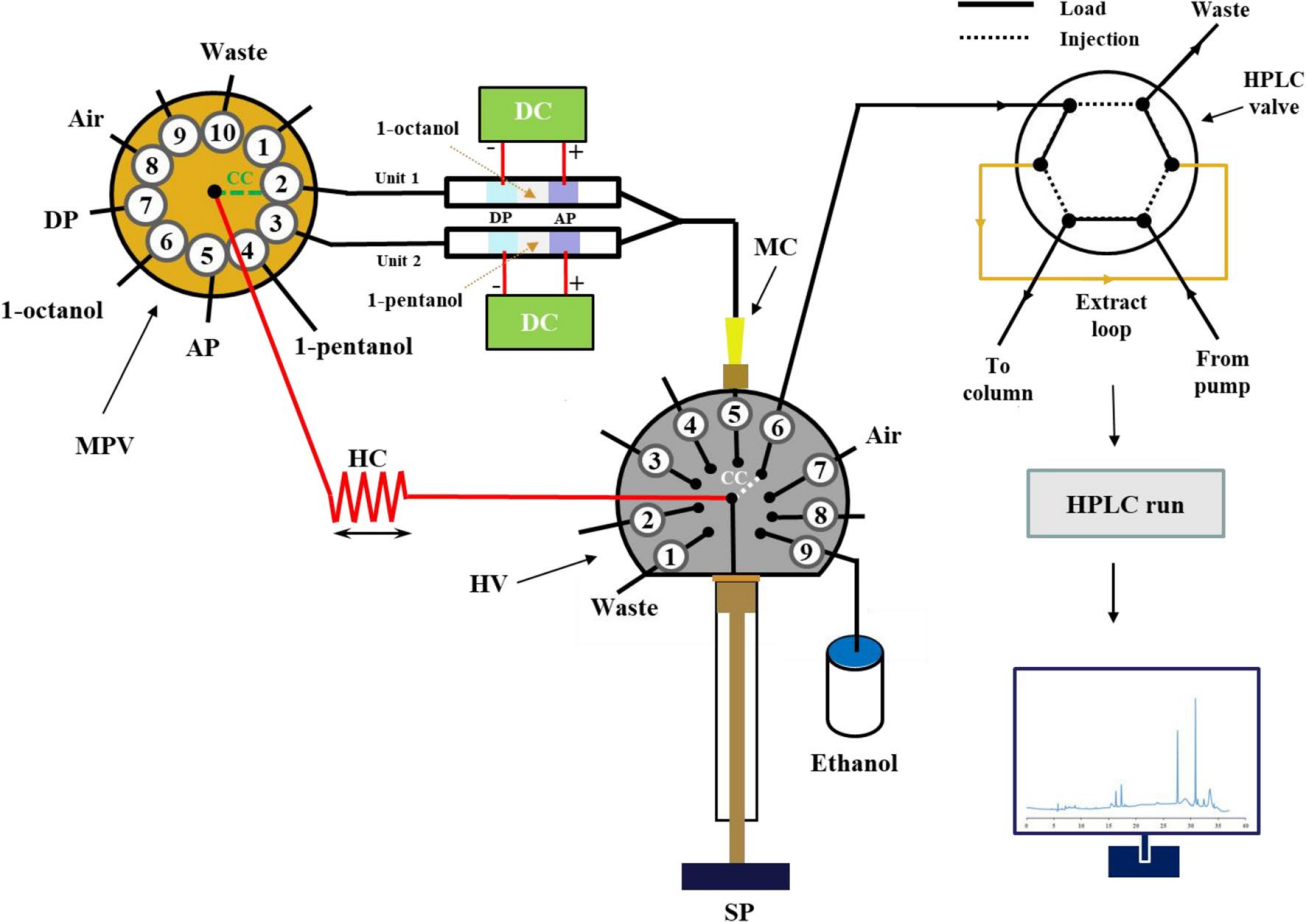
Schematic illustration of the SIA-D-µEME-HPLC-UV-Vis for automatic microextraction/clean-up and detection of PPAs in gastric extracts from bioaccessibility tests. SP, syringe pump; MPV, multi-position valve; MC, mixing cup; HV, head valve; HC, holding coil; CC, central channel; μEME, microelectromembrane extraction; DC, direct current power supply; DP, donor phase; AP, acceptor phase; HPLC, high-performance liquid chromatograph

A diagrammatic representation of the SIA setup for automatic D-μEME with on-line HPLC-UV-Vis detection is shown in Fig. 1. Detailed information of the SIA system including the specifications of the holding coil (HC), the microsyringe pump (SP), the head valve (HV), the multi-position selection valve (MPV), the μEME instrumentation, and the HPLC components are provided in the electronic supplementary information (ESI) file.

For the HPLC separation and detection of the target analytes, eluent A was 100% Milli-Q water, while eluent B was 100% methanol, both containing 1% (v/v) acetic acid. Reversed-phase separations were conducted at 30 °C using a C18 analytical column (250 × 4.6 mm, 5 µm particle size, Lot No. 10-08-M14, Varian, now Agilent) with an integrated C18 guard column. Gradient elution at a flow rate of 0.6 mL/min was carried out as follows: (i) increase from 5 to 65% B over 0–22 min, (ii) increase to 90% B at 23 min, (iii) increase to 100% B at 24 min, (iv) maintain at 100% B for 3 min (24–27 min), (v) decrease to 5% B at 29 min, and finally (vi) equilibration with 5% B at 37 min before the next injection. PPAs were monitored using a photo diode array detection at specific wavelengths: 255 nm for 4-OH-BA, 275 nm for GA and CiA, and 330 nm for ChA and CA. The entire extraction process, including µEME phase formation, activation of the voltage, injection of a measured volume of AP to the HPLC loop, and chromatograph activation, were all automated using the freeware CocoSoft 6.2 [33].

### In-line SI-µEME procedure

The automatic µEME process begins with the sequential delivery of 17 µL of AP, 22 µL of OP, and 17 µL of DP, taken from their respective ports of MPV (#5 for AP, #6 for OP, #7 for DP) to the port #2 of MPV for unit 1 (1-octanol as OP). The HC was then rinsed with 200 µL of ethanol to remove any residual material on its walls and similar steps are repeated for unit 2, except for 1-pentanol as OP from port #4.

Subsequently, two air plugs of 75 µL and 85 µL were introduced behind the previously prepared three phases to position them appropriately between the electrodes for µEME in units 1 and 2, respectively (Fig. 1), upon which the power supplies were activated for 10 min. Once the µEME processes in both units were completed, the EME plugs in unit 2 were pushed first by 60 µL of air, followed by pushing the plugs in unit 1 with 200 µL of air to be able to displace and collect the APs in the mixing cup (MC) attached to the HV (Fig. 1). In this manner, the two APs could be mixed prior to the ensuing HPLC injection, and the DPs and OPs were kept in the units to be retrieved in a further washing step. The collected AP phases were then aspirated from the MC into SP and subsequently pushed to the loop of the HPLC in a heart-cut mode. Table S1 shows the stepwise process of the µEME, handling of the APs, and their injection in the HPLC. Afterwards, both units 1 and 2 were rinsed equally by 100 µL of ethanol from HV (in total 200 µL, pushed into the MC) to remove the remnants of EME phases, followed by 300 µL of air each, whereby the extraction system was prepared for the next run. The MC device was then washed by 100 µL of ethanol from HV twice that were then dispensed to the waste in port #9 of HV. The SP, HC, and HPLC loop were washed with 200 µL of ethanol each at the end of the operational procedure.

### Calculations

The extraction recovery (ER %) of the target analytes by in-line µEME procedure is calculated according to Eq. (1):

1 $$\mathrm{ER}\%=\frac{\mathrm{Peak}\;\mathrm{area}\;\mathrm{of}\;\mathrm{AP}\;\mathrm{after}\;\mathrm{extraction}\;\mathrm{at}\;20\mathrm{mg}/\mathrm L}{\mathrm{Peak}\;\mathrm{area}\;\mathrm{of}\;\mathrm{standard}\;\mathrm{solution}\;\mathrm{at}\;20\mathrm{mg}/\mathrm L}\times100$$

The relative recovery (RR%) is determined from spiked samples following Eq. (2):

2 $$\mathrm{RR}\%=\left[\left({\mathrm C}_{\mathrm{found}}-{\mathrm C}_{\mathrm{real}}\right)/{\mathrm C}_{\mathrm{added}}\right]\times100$$

where *C*_found_ and *C*_real_ are the total concentration found and the concentration of incurred analyte in the real sample, respectively, as obtained from D-μEME using matrix-matched calibration, and *C*_added_ is the concentration of spiked analyte in the real sample.

## Results and discussion

### Configuration of the flow system as a “front end” to HPLC

Preliminary tests were conducted for proper design and arrangement of the SIA network coupled to HPLC analysis. Originally, only MPV was used for the µEME process in combination with a two-way solenoid valve with one outlet for the isolation of the APs into the HPLC loop and the other connected to waste. However, due to significant back pressure from the solenoid and HPLC valves, inconsistent and inaccurate volumes of µEME plugs were formed and also retrieved in each experiment. It was concluded that employing an MC attached to HV as an interface between the SIA setup and the HPLC, in which the APs are dropped in, would tackle the above-mentioned issues. However, when a 1-mL SP volume was used, the AP extracts were not injected into the loop because most of the volume remained in the SP dead volume. To solve this issue, 250 and 100 µL microsyringes were tested instead. The experimental results proved the 100-µL syringe to behave the best as its narrow diameter facilitated the dead volume to be isolated from the AP by an air plug, as a consequence of capillary effects. However, smooth aspiration and delivery by SP of a measured volume of AP (viz., 25 µL) were also necessary; that is, low flow rates (100 µL/min) permitted the APs keeping their integrity with the subsequent straightforward injection into the HPLC, while higher flow rates would still result in the partial or complete retention of the AP volume within the SP dead volume. In conclusion, a 100-µL bidirectional syringe pump operated at 100 µL/min was chosen for the injection step of the collected APs to the HPLC system.

### Operational, chemical, and hydrodynamic parameters of the automatic μEME procedure

DPs and APs pH values were adopted from a previous membrane-based EME of PPAs, that is, 7.0 and 12.0, respectively [13]. These pH values ensure that analytes are negatively charged throughout the entire process as it is required for the electrical-driven extraction across the OP. The volumes of AP and DP were selected to be 17 µL each as the handling and retrieving steps could be performed reliably by the SIA setup. For the OPs, 22 μL of the organic solvents was dispensed to be able to generate similar plug sizes as those of the APs and DPs, because minute amounts of the organic solvents form wetting films onto the walls of the PTFE tubing and therefore, they do not reach the µEME units.

#### Effect of the organic solvents on the μEME procedure

Mass transfer and selectivity are significantly controlled by the composition of the organic plug. The organic phase also affects the stability of the non-supported μEME system by determining the electric current level generated during the extraction process. In the case of acidic analytes, an ideal solvent to be used as the OP should provide moderate dipolarity-polarizability, high hydrogen bond acidity, and low hydrogen bond basicity. Long-chain aliphatic alcohols have proven to be the most suitable choices for those organic compounds according to the literature [34]. Various aliphatic alcohols, such as 1-pentanol, 1-hexanol, 1-heptanol, 1-octanol, and 1-nonanol, as well as DES and a mixture of ionic liquid (Aliquat® 336) as a carrier in alcoholic solvents were herein investigated. In all cases, the DP was ionic strength adjusted with 120 mmol/L of NaCl in the mimicry of the salt content of the pH-adjusted USP gastric fluid, while containing the target compounds at the 20 mg/L level.

**Fig. 2:**
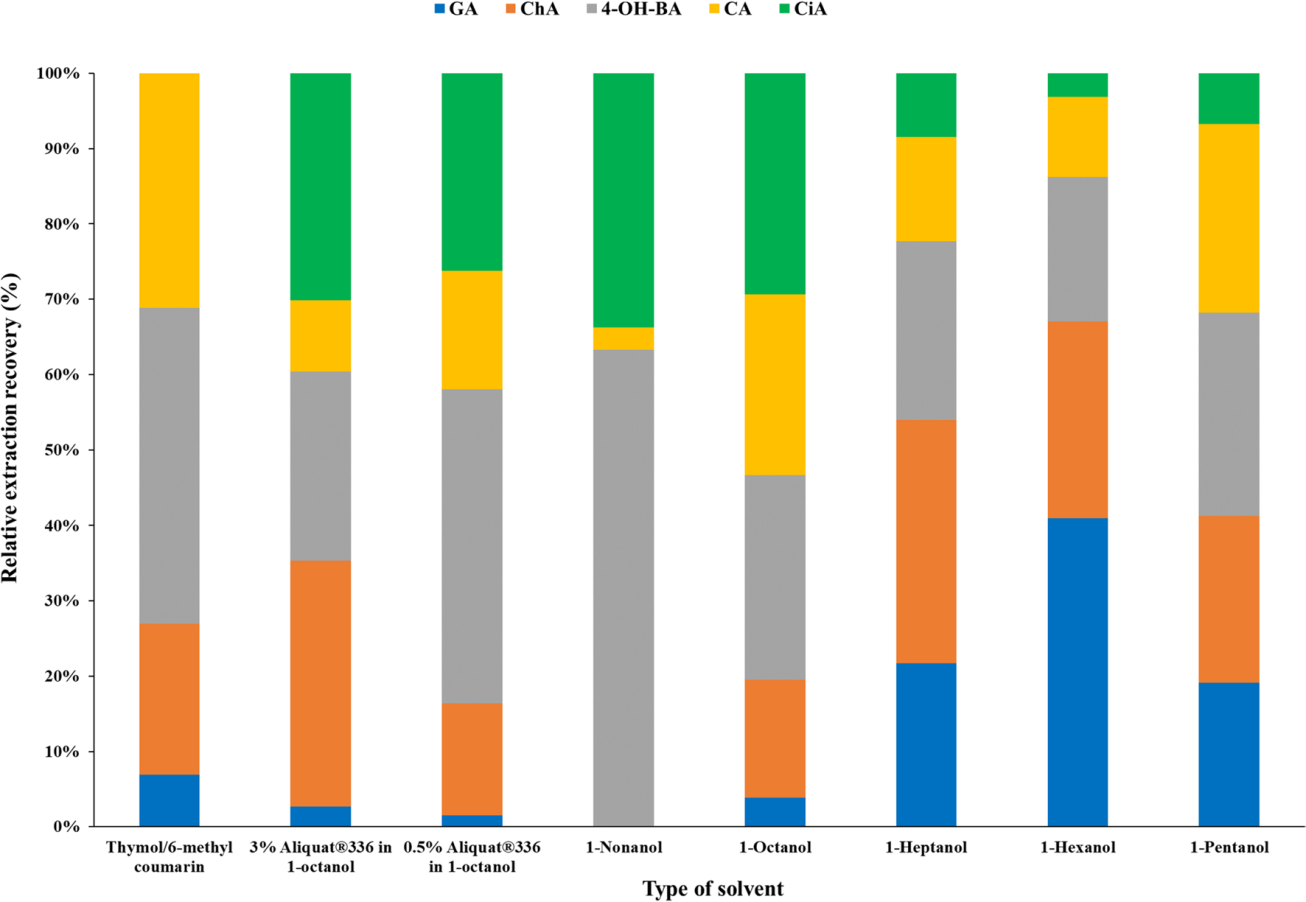
The effect of the OP nature on the performance of the µEME of PPAs (20 mg/L) in 120 mmol/L NaCl for 10 min using maximum voltages that enabled EME phase stability in the SIA system

According to the results depicted in Fig. 2, normalized extraction efficiencies of PPAs vary significantly with the chain length and polarity of the solvent. The most polar solvents like 1-pentanol and 1-hexanol are suitable for extracting GA, whereas this PPA is negligibly extracted by 1-nonanol or 1-octanol. Conversely, the extraction of CiA, the most nonpolar analyte herein studied, is most efficiently extracted with long-chain alcohols like 1-octanol and 1-nonanol. Interestingly, the addition of distinct % of ionic liquid (Aliquat® 336) as a carrier did not improve the overall transfer of the polar target analytes, possibly due to the exchange of chloride ions from the ionic strength adjusted solution, i.e., NaCl, rather than the target analytes. Furthermore, extraction with pure Aliquat® 336 as a solvent was deemed inappropriate due to its high viscosity to be manipulated throughout the flow system. DES features considerable selectivity towards 4-OH-BA, but the DES components seemed to significantly interfere with the CiA detection, making its quantification unreliable. This problem can be, however, solved using HPLC with mass spectrometric detection in future application studies.

Considering the stability of 1-octanol for µEME, its efficiency in extracting medium to high LogP compounds, especially CA, and easy cleaning of the wetting film in the flow network and the effectiveness of 1-pentanol for extraction of lower to medium LogP analytes and its compatibility for automatic handling with the flow system, both solvents were fully leveraged for designing the D-µEME platform, ensuring the comprehensiveness of the dual EME method for extraction of the overall five analytes with acceptable average ER%. It is notable that both solvents could extract medium polarity analytes, such as 4-OH-BA and CA.

#### Effect of the applied voltage and the extraction time on the µEME procedure

**Fig. 3:**
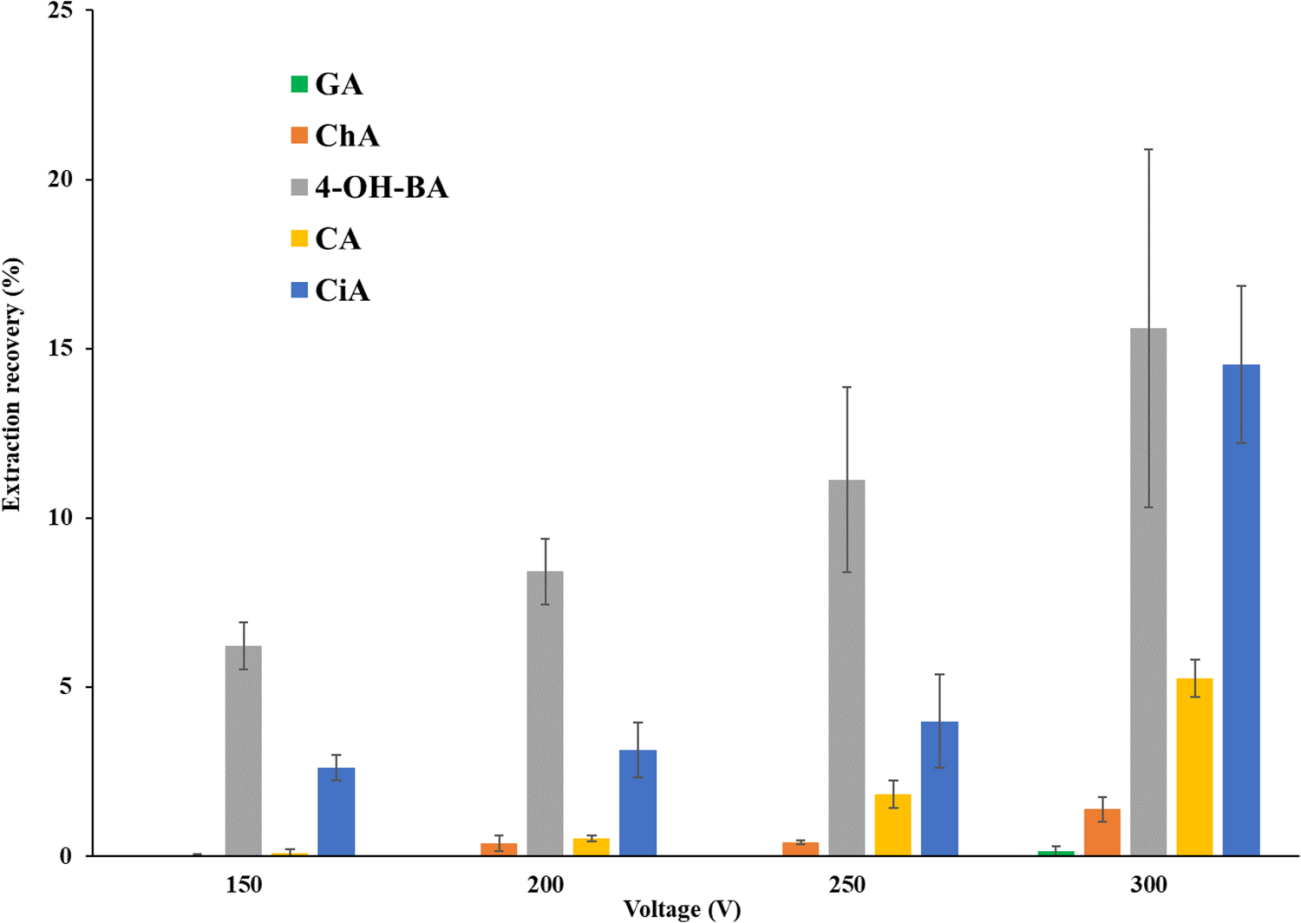
The effect of voltage (150–300 V) on the extraction recovery of 20 mg/L each of the five target PPAs in 120 mmol/L NaCl for an extraction time of 10 min using 1-octanol as OP

DC voltages between 150 and 300 V were studied to assess the impact of the voltage on the extraction efficiency of the target analytes in unit 1 (Fig. 3). When no voltage was applied, no extraction occurred for any analyte in 20 min. However, while increasing the voltage up to 300 V, the extraction efficiency of all of the analytes except GA improved. Under an applied voltage of 300 V, electrical currents were below 20 µA throughout the extraction process using 1-octanol. It is worth noting that despite the application of a high voltage, unit 1, in which 1-octanol was utilized as the solvent, did not encounter any issue regarding the stability of three phases. In the case of unit 2, where 1-pentanol was used as the OP, the highest voltage that could be tolerated under high salinity conditions (using 120 mmol/L of NaCl) was 35 V for 10 min. However, at higher voltages (> 35 V) for 1-pentanol, electrolysis was noticeable and the elevated electrical currents recorded (> 90 µA) rendered the µEME system unstable after 10 min, resulting in phase collapse. In conclusion, voltages of 300 V and 35 V were selected as the most appropriate voltages for units 1 and 2, respectively.

Experiments were conducted to assess the impact of the extraction time within the range of 0 to 20 min for unit 1 on the µEME performance. The results indicated that prolonged extraction times would result in enhancing ER% up to 10 min, beyond which the ER%s of most analytes reached a plateau. Similarly, for unit 2, a maximum of 10-min extraction duration was chosen to ensure three phase stability, while preventing currents in the gastric bioaccessibility tests of the food commodities > 50 µA (Fig. S1) [18].

#### Effect of the ionic strength on the µEME procedure

The performance of the automatic D-µEME method was evaluated for standard solutions of PPAs in the presence and absence of 120 mmol/L NaCl (Fig. S2). The results revealed that ER%s decreased to a large extent in the presence of salt. This decrease could be attributed to the migration of chloride ions, acting as competitors of the target organic acids. 1-Pentanol was significantly affected in the presence of salt, showing an average signal decrease by > fivefold for all the analytes, as compared to > 3.3-fold decrease for octanol. This observation could be explained by the greater permeability of small ions across the more polar 1-pentanol in comparison to 1-octanol. It is notable that the range of extraction recoveries in aqueous solutions, without salt, by µEME (viz., 17.5–40%) using 1-pentanol is comparable to that of a previous study for similar analytes (viz., 26.8–39.9%) using gel-EME [12], but performed manually.

### Analytical performance of the automatic D-µEME method

Under the selected experimental conditions, the analytical performance of the proposed SIA-D-µEME-HPLC method for the target PPAs was evaluated in terms of linearity, limit of detection (LOD), and precision (RSD), as shown in Table 1. Matrix-matched calibration curves were generated using standard solutions at different concentration levels ranging from 1.25 to 80 mg/L prepared in the USP gastric fluid. The calibration curves of peak area against analyte concentration (mg/L) exhibited coefficients of determination ≥ 0.9858. The LOD ((3 × average noise at a given retention time × concentration of the first point in the linear range)/height of the peak of the first concentration point, i.e., S/N = 3) was determined to be in the range of 0.2–3.3 mg/L. The repeatability values (RSD%, *n* = 3) at the concentration of 20 mg/L for each analyte were < 22.4%. The ER% values in pH-adjusted gastric fluid were in the range of 3–7% for the target analytes through D-µEME. As previously mentioned in the “Effect of the ionic strength on the µEME procedure” section, similar to the saline effect, the high ionic strength of the pH-adjusted gastric fluid negatively affects the extraction of the most polar analytes. In fact, previous batchwise EME protocols for PPAs recommended dilution factors in real samples as high as 1:400 and 1:500 to alleviate matrix effects [13], yet the actual ER values were not reported, most likely due to the minute ERs obtained.

**Table 1:** Analytical figures of merit of the automatic SIA-D-µEME-HPLC-UV-Vis method

Analyte	Linear range (mg/L)	Sensitivity (L·mAU/mg)	*R*^2^	LOD (mg/L)	Repeatability (RSD%, *n* = 3)
GA	10–80	0.23	0.9963	3.3	21.0
ChA	2.5–80	2.58	0.9929	0.8	11.2
4-OH-BA	2.5–60	6.66	0.9953	0.6	4.9
CA	5.0–60	6.08	0.9858	1.5	11.4
CiA	1.25–40	5.54	0.9994	0.2	22.4

### Determination of oral bioaccessible PPAs from food commodities

The target PPAs were determined in three different food commodities subjected to in vitro physiologically-based (gastric) extraction tests to ascertain the applicability of the SIA-D-µEME-HPLC-UV-Vis method for oral bioaccessibility tests. To evaluate the trueness of the method, gastric extracts were spiked at the 20 mg/L level for every target PPA (Fig. S3-S5). The RR% for the analytes in the oral bioaccessibility tests ranged from 71.5 to 133.5% with RSDs ranging from 2.6 to 22.7% (Table 2). The electrical current profiles for incurred and spiked samples are shown in the Fig. S1A and S1B for 1-octanol and 1-pentanol, respectively. Currents for the two solvents spanned from 2.4 to 18 µA for 1-octanol and 4.2 to 46 µA for 1-pentanol. These results suggest the suitability and applicability of the proposed automatic D-µEME method in analyzing high-saline samples such as gastric extracts for further bioaccessibility studies of food or environmental samples.

**Table 2:** Trueness of the SIA-D-µEME-HPLC-UV-Vis method as determined by relative recoveries

Sample	Analyte	Spiked (mg/L)	Found (mg/L)	RSD% (*n* = 3)	RR% (*n* = 3)
Coffee bean extract	GA	0	0	**-**	**-**
		20	19.10	18.5	95.5
	ChA	0	18.4	14.9	-
		20	32.8	22.7	72.0
	4-OH-BA	0	5.2	3.1	-
		20	19.5	6.5	71.5
	CA	0	6.8	6.9	-
		20	21.1	17.6	71.5
	CiA	0	0	-	-
		20	22.2	12.5	111
Blueberry	GA	0	0	-	-
		20	20.2	5.6	101
	ChA	0	20.7	20.1	-
		20	40.3	12.7	98.0
	4-OH-BA	0	5.3	2.6	-
		20	24.4	14.0	95.5
	CA	0	6.9	7.4	-
		20	25.7	19.0	94.0
	CiA	0	0	-	-
		20	16.6	4.2	83.0
Eggplant	GA	0	0	-	-
		20	26.7	3.2	133.5
	ChA	0	13.2	18.3	-
		20	35.4	10.7	111
	4-OH-BA	0	5.3	3.5	-
		20	19.9	15.9	73.0
	CA	0	7.3	9.5	-
		20	22.7	14.1	77.0
	CiA	0	0	-	-
		20	23.8	17.4	119

### Greenness study

The AGREEprep metric [35] was selected to evaluate the greenness of the D-µEME-HPLC-UV-Vis method. The inputs for all 10 criteria (see Fig. S6 inset) are provided here: (1) ex situ, (2) 0.054 mL, (3) > 75% of reagents and materials are sustainable or renewable, (4) 1.34 mL or g, (5) 0.044 mL or g, (6) 2 per hour, (7) 2 steps or fewer, fully automated, (8) 120 W, (9) liquid chromatography, and (10) 2 hazard. Our green score (0.56) surpasses those of previously reported liquid and solid phase (micro)extraction approaches for PPAs with green scores spanning from 0.14 to 0.43 [7, 9–11, 36–40], and it is relatively close to previous EME-based systems for polyphenolic compounds (scores of 0.6–0.7) [12, 13], all adapted from the work of Román-Hidalgo et al. [13].

## Conclusion

This paper reports on the first study on the application of miniaturized EME for oral bioaccessibility tests using simulated human gastric fluid. For this purpose, an automatic D-µEME platform employing 1-pentanol (35 V for 10 min) for extracting highly polar and 1-octanol (300 V and 10 min) for less polar PPAs was coupled on-line to HPLC-UV-Vis in a fully automatic extraction and detection setting. The assembled millifluidic D-µEME system was applied to the determination of the target PPAs in three real samples including coffee bean extract, eggplant, and blueberry, providing RR%s in the range of 71.5–133.5% with RSD% ≤ 23%. The developed system is amenable to troublesome samples, like gastric fluids, with very high acidity and salt content (120 mmol/L) with no need of sample dilution. Further work is underway in our lab to couple µEME to HPLC-MS for trace analysis of organic compounds and metabolite thereof in body fluids.

## Supplementary Information

Below is the link to the electronic supplementary material.

Supplementary file1 (DOCX 672 KB)

## References

[1] Da Silva APG, Sganzerla WG, John OD, Marchiosi R. A comprehensive review of the classification, sources, biosynthesis, and biological properties of hydroxybenzoic and hydroxycinnamic acids. Phytochem Rev. 2023. 10.1007/s11101-023-09891-y.

[2] Sobhani M, Farzaei MH, Kiani S, Khodarahmi R. Immunomodulatory; anti-inflammatory/antioxidant effects of polyphenols: a comparative review on the parental compounds and their metabolites. Food Rev Int. 2021;37(8):759–811. 10.1080/87559129.2020.1717523.

[3] Rana A, Samtiya M, Dhewa T, Mishra V, Aluko RE. Health benefits of polyphenols: a concise review. J Food Biochem. 2022;46(10): e14264. 10.1111/jfbc.14264. 35694805 10.1111/jfbc.14264

[4] El-Saadony MT, Yang T, Saad AM, Alkafaas SS, Elkafas SS, Eldeeb GS, Mohammed DM, Salem HM, Korma SA, Loutfy SA, Alshahran MY, Ahmed AE, Mosa WFA, Abd El-Mageed TA, Ahmed AF, Fahmy MA, El-Tarabily MK, Mahmoud RM, AbuQamar SF, El-Tarabily KA, Lorenzo JM. Polyphenols: chemistry, bioavailability, bioactivity, nutritional aspects and human health benefits: a review. Int J Biol Macromol. 2024;277: 134223. 10.1016/j.ijbiomac.2024.134223. 39084416 10.1016/j.ijbiomac.2024.134223

[5] Bergantin C, Maietti A, Cavazzini A, Pasti L, Tedeschi P, Brandolini V, Marchetti N. Bioaccessibility and HPLC-MS/MS chemical characterization of phenolic antioxidants in red chicory (Cichorium intybus). J Funct Foods. 2017;33:94–102. 10.1016/j.jff.2017.02.037.

[6] Raposo F, Borja R, Gutiérrez-González JA. A comprehensive and critical review of the unstandardized Folin-Ciocalteu assay to determine the total content of polyphenols: the conundrum of the experimental factors and method validation. Talanta. 2024;272: 125771. 10.1016/j.talanta.2024.125771. 38394752 10.1016/j.talanta.2024.125771

[7] Saraji M, Mousavinia F. Single-drop microextraction followed by in-syringe derivatization and gas chromatography-mass spectrometric detection for determination of organic acids in fruits and fruit juices. J Sep Sci. 2006;29(9):1223–9. 10.1002/jssc.200500345. 16833079 10.1002/jssc.200500345

[8] Citová I, Sladkovský R, Solich P. Analysis of phenolic acids as chloroformate derivatives using solid phase microextraction–gas chromatography. Anal Chim Acta. 2006;573–574:231–41. 10.1016/j.aca.2006.04.077. 17723529 10.1016/j.aca.2006.04.077

[9] Saraji M, Mousavi F. Use of hollow fibre-based liquid–liquid–liquid microextraction and high-performance liquid chromatography–diode array detection for the determination of phenolic acids in fruit juices. Food Chem. 2010;123(4):1310–7. 10.1016/j.foodchem.2010.06.012.

[10] Khezeli T, Daneshfar A, Sahraei R. A green ultrasonic-assisted liquid–liquid microextraction based on deep eutectic solvent for the HPLC-UV determination of ferulic, caffeic and cinnamic acid from olive, almond, sesame and cinnamon oil. Talanta. 2016;150:577–85. 10.1016/j.talanta.2015.12.077. 26838445 10.1016/j.talanta.2015.12.077

[11] Bakar NBA, Makahleh A, Saad B. In-vial liquid–liquid microextraction-capillary electrophoresis method for the determination of phenolic acids in vegetable oils. Anal Chim Acta. 2012;742:59–66. 10.1016/j.aca.2012.02.045. 22884208 10.1016/j.aca.2012.02.045

[12] Asadi S, Nojavan S, Behpour M, Mahdavi P. Electromembrane extraction based on agarose gel for the extraction of phenolic acids from fruit juices. J Chromatogr B. 2020;1159: 122401. 10.1016/j.jchromb.2020.122401. 10.1016/j.jchromb.2020.122401 33126069

[13] Román-Hidalgo C, López-Pérez G, Villar-Navarro M, Martín-Valero MJ. Green electromembrane extraction procedure based on biodegradable chitosan films for determination of polyphenolic compounds in food samples: greenness assessment of the sample preparation approach. Talanta. 2023;253: 124034. 10.1016/j.talanta.2022.124034.

[14] Martins RO, de Araújo GL, Simas RC, Chaves AR. Electromembrane extraction (EME): fundamentals and applications. Talanta Open. 2023;7: 100200. 10.1016/j.talo.2023.100200.

[15] Eie LV, Pedersen-Bjergaard S, Hansen FA. Electromembrane extraction of polar substances – status and perspectives. J Pharm Biomed Anal. 2022;207: 114407. 10.1016/j.jpba.2021.114407. 34634529 10.1016/j.jpba.2021.114407

[16] Hansen FA, Petersen NJ, Kutter JP, Pedersen-Bjergaard S. Electromembrane extraction in microfluidic formats. J Sep Sci. 2022;45(1):246–57. 10.1002/jssc.202100603. 34562339 10.1002/jssc.202100603

[17] Li J, Zhu R, Shen X, Huang C. Functional materials and chemicals in electromembrane extraction. TrAC Trends Anal Chem. 2022;150:116574. 10.1016/j.trac.2022.116574.

[18] Zhou C, Dowlatshah S, Hay AO, Schüller M, Pedersen-Bjergaard S, Hansen FA. Generic liquid membranes for electromembrane extraction of bases with low or moderate hydrophilicity. Anal Chem. 2023;95(23):8982–9. 10.1021/acs.analchem.3c01052. 37259537 10.1021/acs.analchem.3c01052 PMC10267886

[19] Drouin N, Kubáň P, Rudaz S, Pedersen-Bjergaard S, Schappler J. Electromembrane extraction: overview of the last decade. TrAC Trends Anal Chem. 2019;113:357–63. 10.1016/j.trac.2018.10.024.

[20] Kubáň P, Boček P. Simultaneous micro-electromembrane extractions of anions and cations using multiple free liquid membranes and acceptor solutions. Anal Chim Acta. 2016;908:113–20. 10.1016/j.aca.2016.01.007. 26826693 10.1016/j.aca.2016.01.007

[21] Dvořák M, Seip KF, Pedersen-Bjergaard S, Kubáň P. Semi-automated set-up for exhaustive micro-electromembrane extractions of basic drugs from biological fluids. Anal Chim Acta. 2018;1005:34–42. 10.1016/j.aca.2017.11.081. 29389317 10.1016/j.aca.2017.11.081

[22] Javier Carrasco-Correa E, Kuban P, Cocovi-Solberg DJ, Miró M. Fully automated electric-field-driven liquid phase microextraction system with renewable organic membrane as a front end to high performance liquid chromatography. Anal Chem. 2019;91(16):10808–15. 10.1021/acs.analchem.9b02453. 31307195 10.1021/acs.analchem.9b02453

[23] Horstkotte B, Miró M, Solich P. Where are modern flow techniques heading to? Anal Bioanal Chem. 2018;410:6361–70. 10.1007/s00216-018-1285-2. 30083907 10.1007/s00216-018-1285-2

[24] Miró M, Hansen EH. Recent advances and future prospects of mesofluidic lab-on-a-valve platforms in analytical sciences–a critical review. Anal Chim Acta. 2012;750:3–15. 10.1016/j.aca.2012.03.049. 23062425 10.1016/j.aca.2012.03.049

[25] Sahragard A, Carrasco-Correa EJ, Cocovi-Solberg DJ, Kubáň P, Miró M. Enhancing the concentration capability of nonsupported electrically driven liquid-phase microextraction through programmable flow using an all-in-one 3D-printed optosensor: a proof of concept. Anal Chem. 2024;96(27):11068–75. 10.1021/acs.analchem.4c02139. 38917332 10.1021/acs.analchem.4c02139 PMC11238157

[26] Šlampová A, Kubáň P. Two-phase micro-electromembrane extraction across free liquid membrane for determination of acidic drugs in complex samples. Anal Chim Acta. 2019;1048:58–65. 10.1016/j.aca.2018.10.013. 30598158 10.1016/j.aca.2018.10.013

[27] Šlampová A, Kubáň P. Two-phase micro-electromembrane extraction with a floating drop free liquid membrane for the determination of basic drugs in complex samples. Talanta. 2020;206: 120255. 10.1016/j.talanta.2019.120255. 31514842 10.1016/j.talanta.2019.120255

[28] Sahragard A, Dvořák M, Pagan-Galbarro C, Carrasco-Correa EJ, Kubáň P, Miró M. 3D-printed stereolithographic fluidic devices for automatic nonsupported microelectromembrane extraction and clean-up of wastewater samples. Anal Chim Acta. 2024;342362. 10.1016/j.aca.2024.342362. 10.1016/j.aca.2024.342362 38438239

[29] Sahragard A, Dvorak M, J. Carrasco-Correa E, Varanusupakul P, Kuban P, Miró M. Programmable millifluidic platform integrating automatic electromembrane extraction cleanup and in-line electrochemical detection: a proof of concept. ACS Sens. 2022;7(10):3161-3168. 10.1021/acssensors.2c01648. 10.1021/acssensors.2c01648 PMC9623577 36200176

[30] Kuban P. Salt removal from microliter sample volumes by multiple phase microelectromembrane extractions across free liquid membranes. Anal Chem. 2017;89(16):8476–83. 10.1021/acs.analchem.7b02017. 28722395 10.1021/acs.analchem.7b02017

[31] Hay AO, Trones R, Herfindal L, Skrede S, Hansen FA. Determination of methotrexate and its metabolites in human plasma by electromembrane extraction in conductive vials followed by LC-MS/MS. Adv Sample Prep. 2022;2: 100011. 10.1016/j.sampre.2022.100011.

[32] United States Pharmacopeial Convention. The United States Pharmacopeia: USP 27; The National Formulary: NF 22. Rockville, MD; 2024.

[33] Cocovi-Solberg DJ, Miró M. CocoSoft: educational software for automation in the analytical chemistry laboratory. Anal Bioanal Chem. 2015;407(21):6227–33. 10.1007/s00216-015-8834-8. 26143060 10.1007/s00216-015-8834-8

[34] Huang C, Gjelstad A, Pedersen-Bjergaard S. Organic solvents in electromembrane extraction: recent insights. Rev Anal Chem. 2016;35(4):169–83. 10.1515/revac-2016-0008.

[35] Wojnowski W, Tobiszewski M, Pena-Pereira F, Psillakis E. AGREEprep – analytical greenness metric for sample preparation. TrAC Trends Anal Chem. 2022;149:116553. 10.1016/j.trac.2022.116553.

[36] Xue Y, Xu X-S, Yong L, Hu B, Li X-D, Zhong S-H, Li Y, Xie J, Qing L-S. Optimization of vortex-assisted dispersive liquid-liquid microextraction for the simultaneous quantitation of eleven non-anthocyanin polyphenols in commercial blueberry using the multi-objective response surface methodology and desirability function approach. Molecules. 2018;23(11):2921. 10.3390/molecules23112921. 30423914 10.3390/molecules23112921 PMC6278316

[37] Campone L, Piccinelli AL, Pagano I, Carabetta S, Di Sanzo R, Russo M, Rastrelli L. Determination of phenolic compounds in honey using dispersive liquid–liquid microextraction. J Chromatogr A. 2014;1334:9–15. 10.1016/j.chroma.2014.01.081. 24565235 10.1016/j.chroma.2014.01.081

[38] Yang P, Li H, Wang H, Han F, Jing S, Yuan C, Guo A, Zhang Y, Xu Z. Dispersive liquid-liquid microextraction method for HPLC determination of phenolic compounds in wine. Food Anal Methods. 2017;10:2383–97. 10.1007/s12161-016-0781-2.

[39] Li Y, He Z, Bao Y, Zhu Q, Ning Y, Tian Z. Zhu X (2022) Simultaneous determination of fifteen polyphenols in fruit juice using ultrahigh-performance liquid chromatography-tandem mass spectrometry combining dispersive liquid-liquid microextraction. Int J Anal Chem. 2022;1:5486290. 10.1155/2022/5486290. 10.1155/2022/5486290 PMC8967586 35371261

[40] Citová I, Sladkovský R, Solich P. Analysis of phenolic acids as chloroformate derivatives using solid phase microextraction–gas chromatography. Anal Chim Acta. 2006;573:231–41. 10.1016/j.aca.2006.04.077. 17723529 10.1016/j.aca.2006.04.077

