# Supplementary material for: Dual microelectromembrane extraction as a tunable platform for the determination of antioxidant compounds with varied hydrophobicity in oral bioaccessibility assays of food commodities: a proof of concept
Source: Anal Bioanal Chem. 2025 Feb 1;417(7):1421–30. doi: 10.1007/s00216-025-05744-z (PMC11861116; doi:10.1007/s00216-025-05744-z)

**Supporting Information**

**Dual microelectromembrane extraction as a tunable platform for the determination of antioxidant compounds with varied hydrophobicity in oral bioaccessibility assays of food commodities: A proof of concept**

Ali Sahragard^*a^, Carlos Pagan-Galbarro^a^, David J Cocovi-Solberg^b^, Manuel Miró^*a^

^a^FI-TRACE Group, Department of Chemistry, Faculty of Science, University of the Balearic Islands, Carretera de Valldemossa km 7.5, E-07122 Palma de Mallorca, Illes Balears, Spain.

^b^University of Natural Resources and Life Sciences, Vienna, Department of Chemistry, Institute of Analytical Chemistry, Muthgasse 18, 1190, Vienna, Austria

**10 pages**

*Corresponding authors. E-mails: [ali.sahragard@uib.es](mailto:ali.sahragard@uib.es), [manuel.miro@uib.es](mailto:manuel.miro@uib.es)

**Flow system and HPLC apparatus.** The sequential injection analysis (SIA) system encompasses a 30 mm-stroke bidirectional microsyringe pump (SP) equipped with a 100 μL-borosilicate glass syringe from Cavro Scientific Instruments (San Jose, CA, USA) and a 10-port multi-position selection valve (MPV, model VICI Cheminert 11X-0231L) from Valco Instruments Co. Inc (Schenkon, Switzerland). The SP ceramic 9-position head valve (HV) connects the SP to the MPV using a 15 cm-long holding coil (HC) made of PTFE tubing (1.5 mm ID, 2.4 mm OD, Avantor, Llinars del Vallès, Spain) and to the HPLC through port #6 (see Fig. 1 in the main text). The external ports (1–10) of MPV are computer-controlled via the central channel (CC) for automatic handling of all liquids. For μEME experiments, two 4 cm-long PTFE tubing (2.5 mm ID) connected to two transfer lines (1.5 mm ID, 2.4 mm OD PTFE tubing) attached to ports #2 and #3 of MPV are used as the in-line μEME units (see Fig. 1). The µEME units are perforated to accommodate two 2 cm-long platinum wires (0.25 mm-thick, 99.9%, Thermo Scientific, Madrid, Spain), working as electrodes, separated 6 mm each other. These electrodes are then fixed to the tubing using a drop of the 3D printer clear resin FLGPCL02 (Formlabs, Somerville, USA) following curation in a UV oven for 30 min at 5000 W (APPLIGENE system, CL-1000 model from Analytik Jena US LLC, Upland, Canada). The outlets of the two µEME units are connected with each other with a Y-shaped polymeric connector (see Fig. 1) that enable transferring the mixed APs to a yellow 200 µL-micropipette tip (called mixing cup in the main text) mounted on the position 5 of the HV using a ¼-28 nut and a ferrule. 1.5 mL Eppendorf vials containing µEME solutions and samples were connected to the flow system through PTFE tubing.

Two power supplies (ES 0300-0.45, Delta Elektronika, Zierikzee, The Netherlands) were used for performing in-line μEME experiments automatically. The power supplies were able to provide a current output of 0–0.45 mA and a voltage output of 0–300 V. Additionally, the power supplies were coupled to 72–7730A electronic multimeters (Tenma Test Equipment, Chicago, IL, USA) to monitor the electric current profiles. For μEME, the negative and positive electrodes were fixed to the tubing platform in the position of the DP and the AP, respectively.

An HPLC system (Jasco, Tokyo, Japan) controlled by Jasco’s ChromNAV 2.0 was used for the chromatographic analyses. The HPLC setup is composed of (i) PU-4180 RHPLC pump (enduring up to 700 bar), (ii) AS-4050 HPLC autosampler equipped with a high pressure injection valve furnished with a sample loop of 25 μL (0.5 mm ID PEEK tubing) (iii) GECKO 2000 column heater for the analytical column, and (iv) MD-4017 photodiode array detector. Peak area was used as the analytical readout.


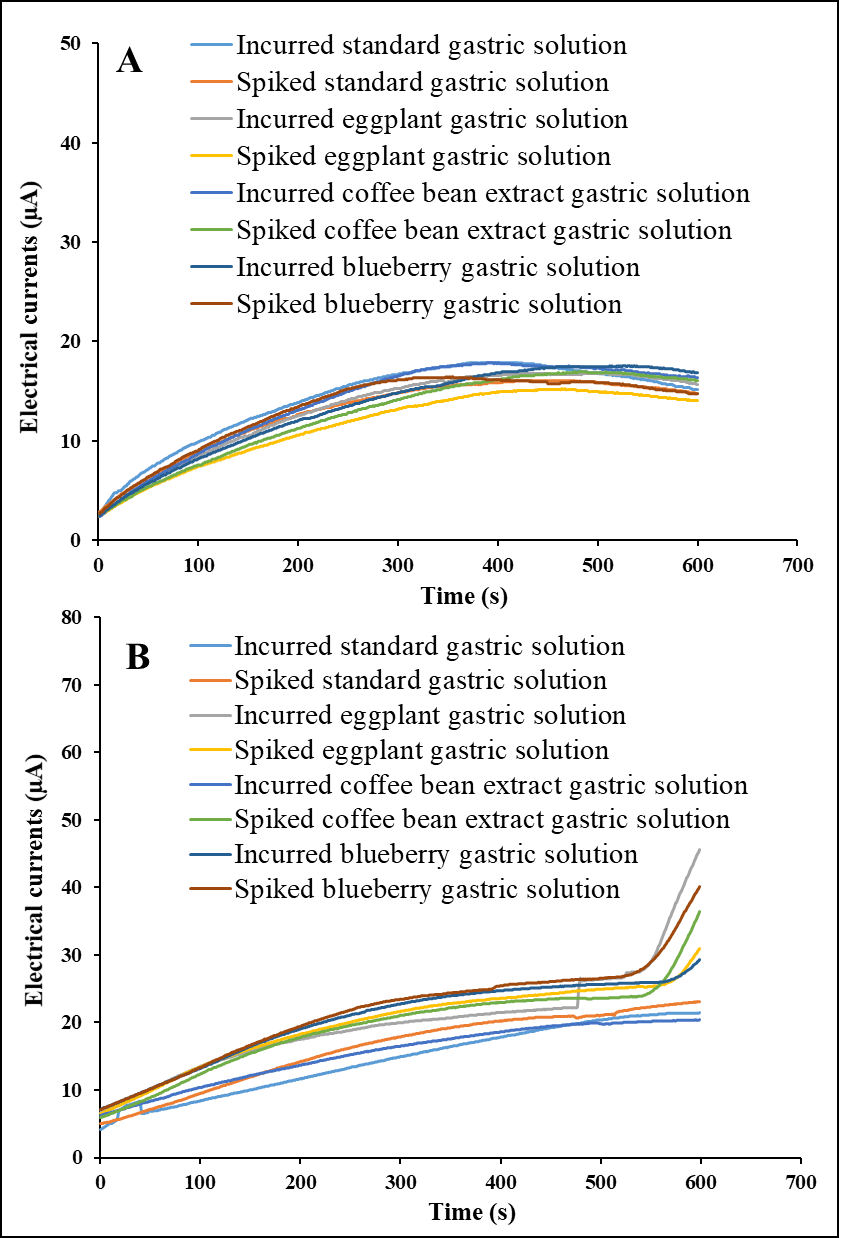


**Fig. S1.** Electrical current profiles of the gastric bioaccessibility assays of PPAs in various real samples as obtained by automatic SIA-D-µEME-HPLC-UV-Vis: A) using 1-octanol under an extraction voltage of 300 V, extraction time of 10 min in unit 1 and B) 1-pentanol under an extraction voltage of 35 V; extraction time of 10 min under platform 2. Spiked standard/samples at the 20 mg/L level of the five target PPAs.


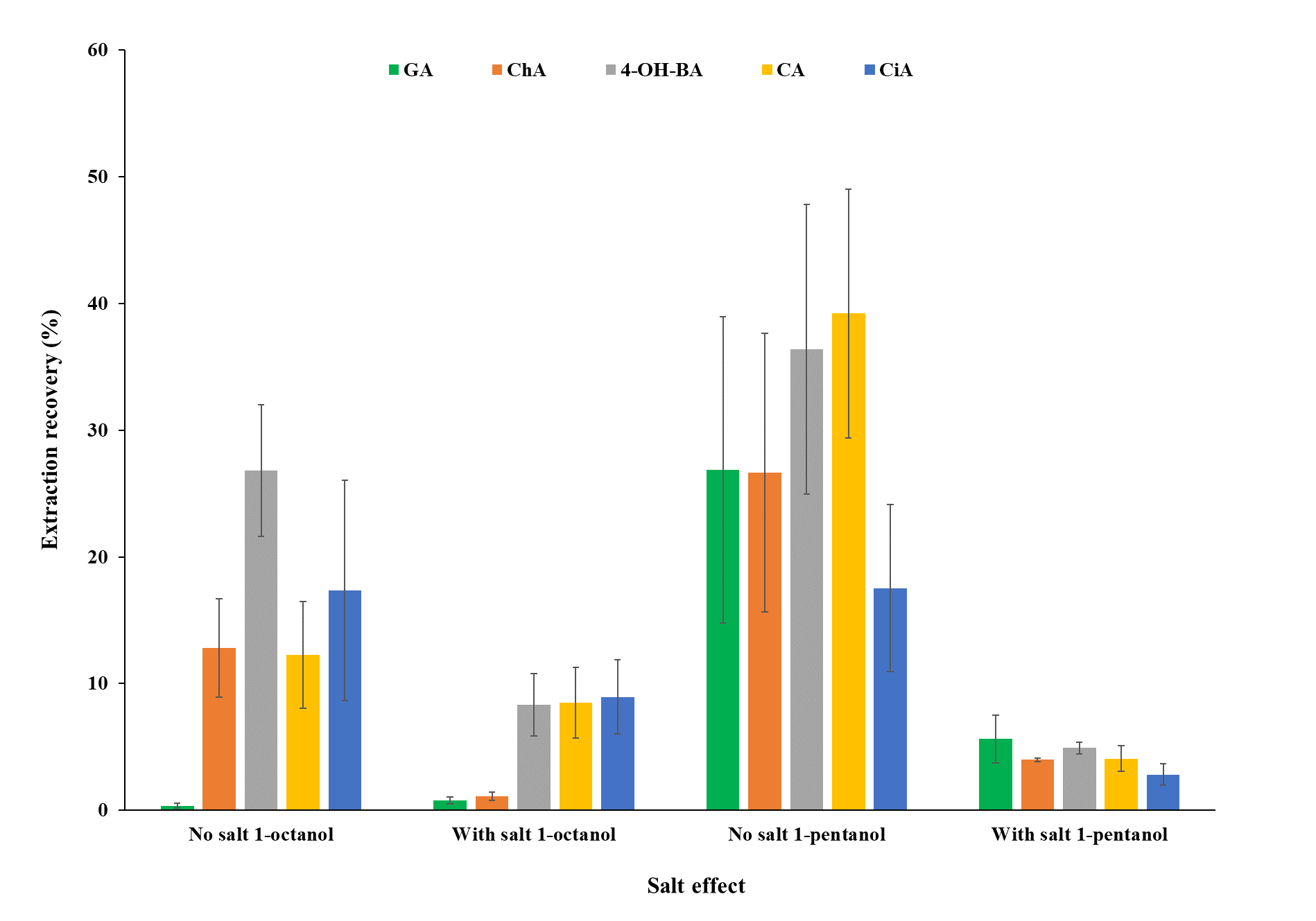


**Fig. S2**. The effect of ionic strength (120 mmol/L NaCl) onto the µEME performance. Experimental conditions: 10 min of extraction time, 20 mg/L (each) of mix PPAs, and 300 V for 1-octanol and 35 V for 1-pentanol.

**
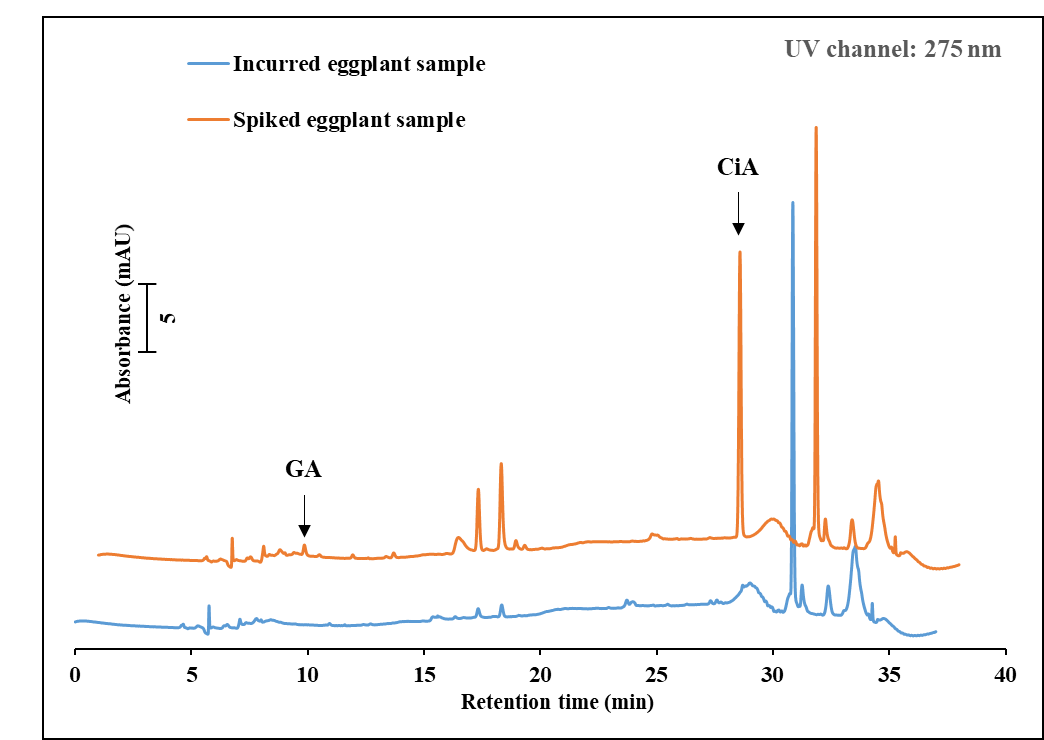
**

**Fig. S3.** Chromatogram for detection of GA and CiA at 275 nm as obtained after SIA-D-µEME-HPLC-UV-Vis for the incurred eggplant sample (blue) and spiked eggplant sample at the 20 mg/L level (brown). Note: the chromatograms were shifted in the X and/or Y-axis directions for better readability.

**
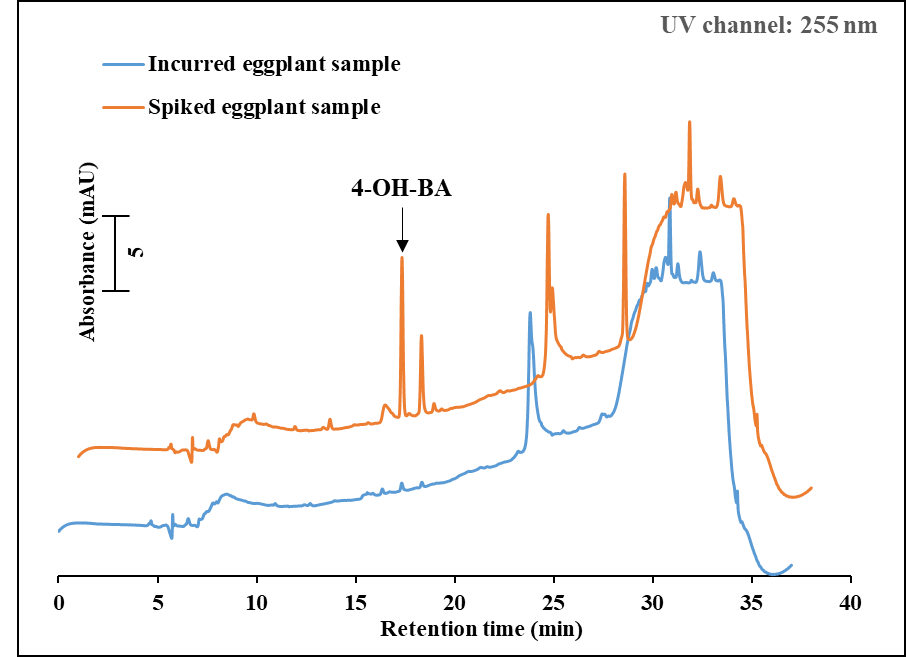
**

**Fig. S4.** Chromatogram for detection of 4-OH-BA at 255 nm as obtained after SIA-D-µEME-HPLC-UV-Vis procedure for the incurred eggplant sample (blue) and spiked eggplant sample at the 20 mg/L level (brown). Note: the chromatograms were shifted in the X and/or Y-axis directions for better readability.

**
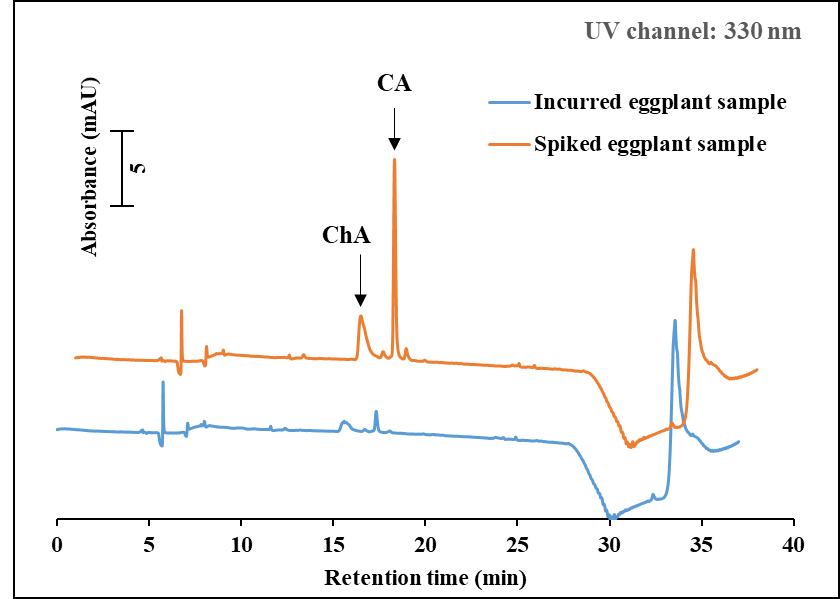
**

**Fig. S5.** Chromatogram for detection of ChA and CA at 330 nm as obtained after SIA-D-µEME-HPLC-UV-Vis for the incurred eggplant sample (blue) and spiked eggplant sample at the 20 mg/L level (brown). Note: the chromatograms were shifted in the X and/or Y-axis directions for better readability.

**B**


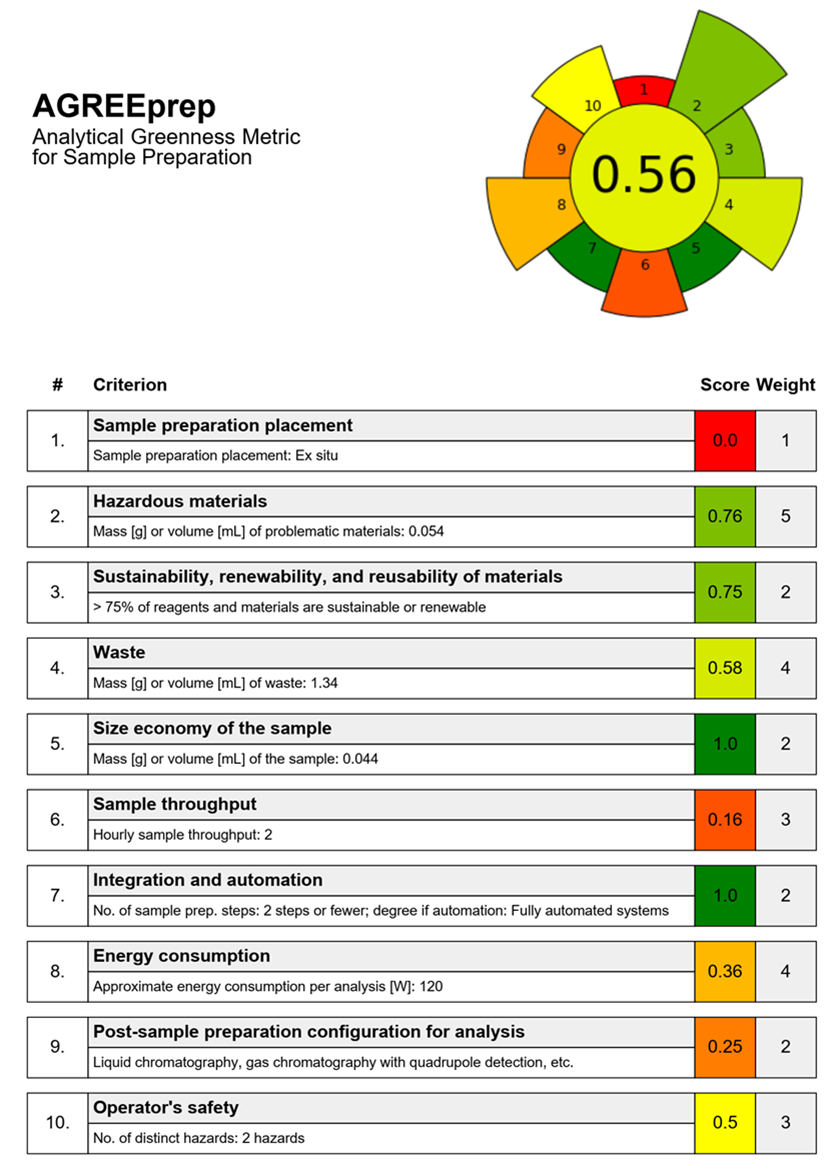


**Fig. S6.** Evaluation of the automatic SIA-D-µEME-HPLC-UV-Vis greenness by application of the AGREEprep tool.

**Table S1.** Summary of the operational procedure for the automatic SIA-D-µEME-HPLC-UV-Vis for the detection of PPAs in gastric extracts of food commodities.


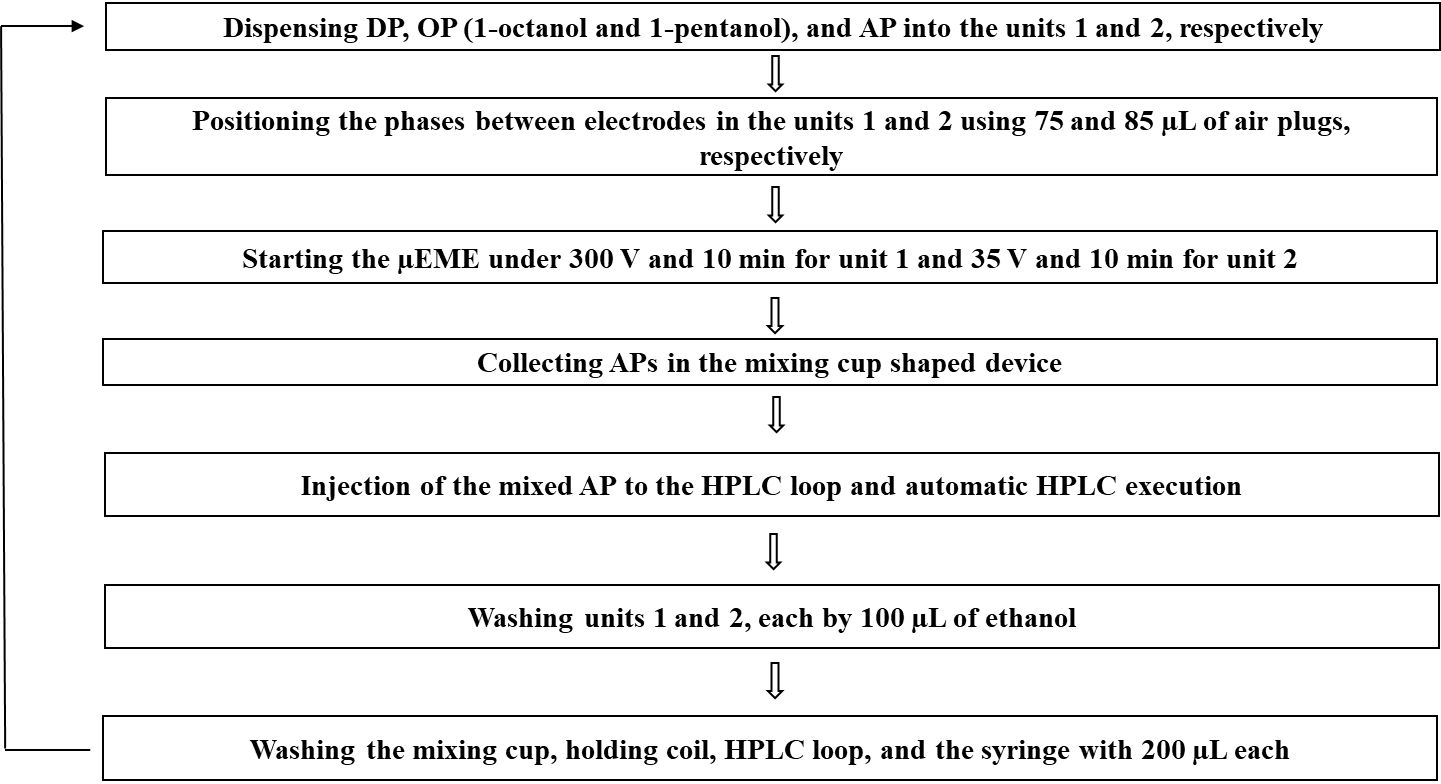

Supplement: Supplementary file 1 — Supplementary file1 (DOCX 672 KB) [file 216_2025_5744_MOESM1_ESM.docx]
